# Quantitative Profiling of the Lymph Node Clearance Capacity

**DOI:** 10.1038/s41598-018-29614-0

**Published:** 2018-07-26

**Authors:** Cristina C. Clement, Wei Wang, Monika Dzieciatkowska, Marco Cortese, Kirk C. Hansen, Aniuska Becerra, Sangeetha Thangaswamy, Irina Nizamutdinova, Jee-Young Moon, Lawrence J. Stern, Anatoliy A. Gashev, David Zawieja, Laura Santambrogio

**Affiliations:** 1 Department of Pathology, Albert Einstein College of Medicine, 1300 Morris Park Avenue, New York, NY 10461 USA; 2 Department of Epidemiology & Population Health, Albert Einstein College of Medicine, 1300 Morris Park Avenue, New York, NY 10461 USA; 3 Department of Medical Physiology, Texas A&M Health Science Center, 702 SW HK Dodgen Loop, Temple, TX 76504 USA; 4 Department of Biochemistry and Molecular Genetics, University of Colorado Denver 12801 E 17th Ave, Aurora, CO 80045 USA; 5 Department of Pathology, University of Massachusetts Medical School, 368 Plantation St, Worcester, MA 01605 USA

## Abstract

Transport of tissue-derived lymphatic fluid and clearance by draining lymph nodes are pivotal for maintenance of fluid homeostasis in the body and for immune-surveillance of the self- and non-self-proteomes. Yet a quantitative analysis of nodal filtration of the tissue-derived proteome present in lymphatic fluid has not been reported. Here we quantified the efficiency of nodal clearance of the composite proteomic load using label-free and isotope-labeling proteomic analysis of pre-nodal and post-nodal samples collected by direct cannulation. These results were extended by quantitation of the filtration efficiency of fluorophore-labeled proteins, bacteria, and beads infused at physiological flow rates into pre-nodal lymphatic collectors and collected by post-nodal cannulation. We developed a linear model of nodal filtration efficiency dependent on pre-nodal protein concentrations and molecular weight, and uncovered criteria for disposing the proteome incoming from defined anatomical districts under physiological conditions. These findings are pivotal to understanding the maximal antigenic load sustainable by a draining node, and promote understanding of pathogen spreading and nodal filtration of tumor metastasis, potentially helping to improve design of vaccination protocols, immunization strategies and drug delivery.

## Introduction

Blood circulating throughout the capillary bed is not in direct contact with the cellular layers of each parenchymal tissue. Thus lipids, proteins, and small molecules need to extravasate in order to provide cellular nutrients and to hydrate tissue cells^1,2^. Extravasation is mediated by hydrostatic pressure inside the blood capillaries and by the Starling forces that drive the ultrafiltration process, moving proteins, macromolecules, and associated water into the interstitial space. A fraction of the extravasated fluid will be absorbed back into the capillary bed, but most will remain in the interstitial tissue^1,2^. These products of capillary extravasation, combined with secreted products deriving from cellular metabolism and catabolism, make up the interstitial fluid that baths every parenchymal organ^3^. Under physiological conditions, in humans, around 8–10 liters of interstitial fluid are formed daily, which need to be returned to the blood circulation to prevent tissue edema^1^. However, the vast majority of the interstitial fluid will not directly be reabsorbed into the blood system, but rather will be collected into the lymphatic capillaries as lymph, and will pass through one or more of the 600–800 draining lymph nodes disseminated throughout the human body, before circulating into the thoracic duct and then the vena cava^2^.

There are several possible explanations why interstitial fluid does not drain directly into the general blood circulation but instead is filtered through the lymph nodes. First, lymphatic passage through the nodes ensures that tissue-invading pathogens do not directly enter into the bloodstream but can be captured by dendritic cells and macrophages residing in the lymph node. Second, the collection of products of tissue remodeling, cellular secretion/processing, and extracellular debris by lymphatic fluid ensures that nodal immune cells are constantly exposed to the self-proteome from each parenchymal organ, helping to maintain peripheral tolerance^2,4–7^. Third, immune cells patrolling peripheral tissues can use lymph flow as a fast and direct conduit to lymph nodes. Fourth, lymph composition at different times and locations can vary widely in protein concentration, electrolytes composition, pH and cellular composition, as opposed to blood, where these parameters are tightly controlled. Thus, the lymph, as observed in both physiological and pathological conditions, can withstand changes occurring in the interstitial fluid without compromising body homeostasis, and can act as a buffer between peripheral tissues and the blood circulatory system^2^.

An important question is the efficiency of nodal clearance and the quantitative impact of nodal filtration on fluid balance, homeostasis, and protein/pathogen clearance. In *vivo* analyses of pathogens and antigens trafficking to the lymph nodes have been reported, but overall measurements of nodal clearance capacity for a complex proteome still is missing^4,8–11^. Towards this goal, we have utilized state-of-the-art, label-free quantitative (LFQ) proteomics complemented by a tandem mass tag (TMT) isotope labeling approach to identify the global proteomic changes in the pre- and post-nodal mesenteric lymph collected from healthy rats. In addition we followed lymphatic transport and nodal processing of fluorochrome-labelled, proteins, bacteria and beads, by direct cannulation of pre-nodal lymphatics followed by post-nodal collection and quantification. The picture that emerges is of lymph nodes as very efficient filtration devices, with concentration-dependent filtration efficiency across molecular sizes.

## Results

**Figure 1:**
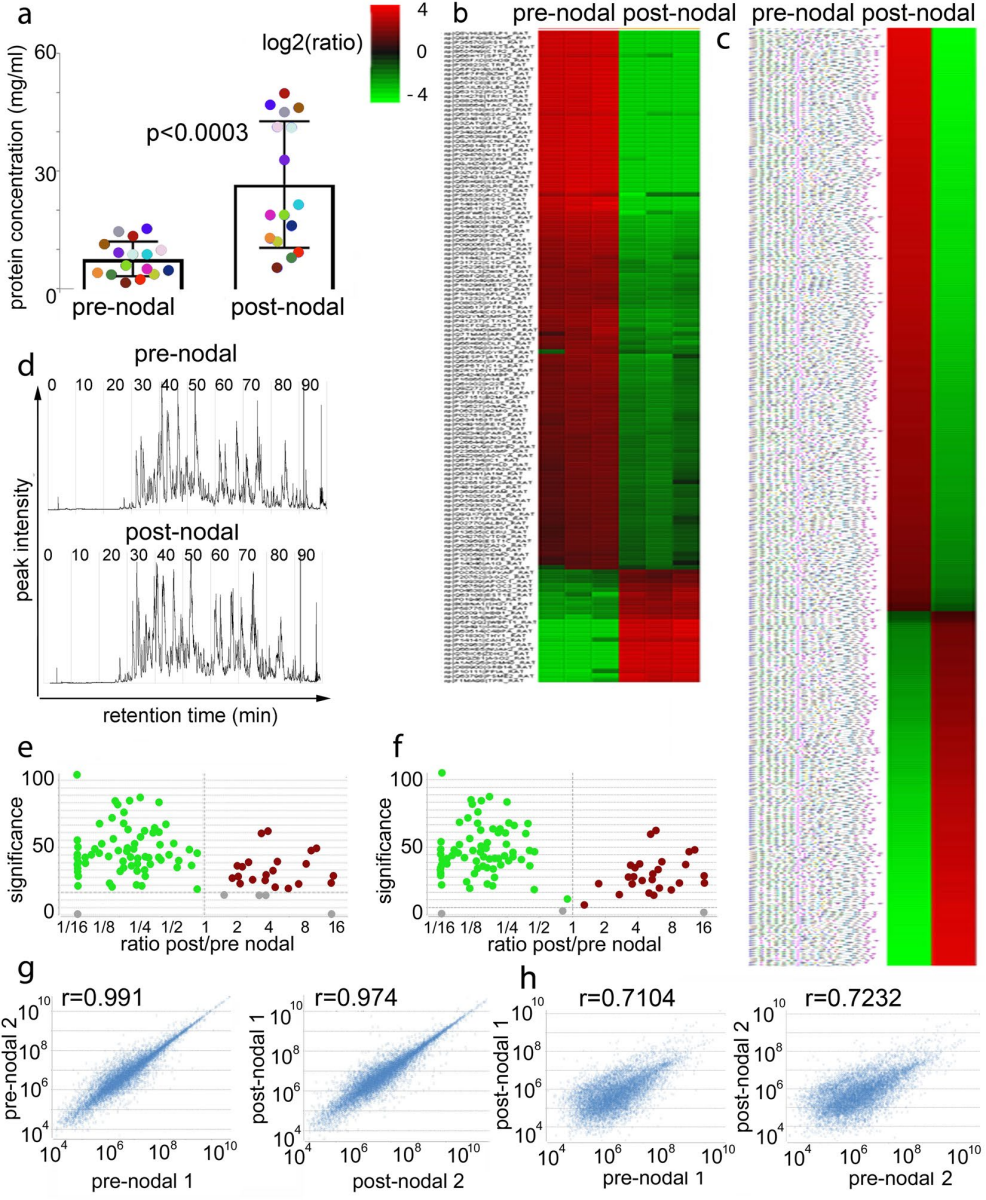
Quantitative proteomic analysis of nodal clearance of afferent lymph. **(A)** Total protein concentration in lymph collected from afferent and efferent lymphatics. Each dot represents protein amount as detected in equal volume of pre and post-nodal lymph collected from 16 individual rats (each color represents an individual animal). Measurements were performed in triplicates for each sample and statistical analysis performed using a two-tailed t-test; average and standard deviation are reported. **(b**,**c)** Heat maps of representative biological and technical triplicates of pre- and post-nodal proteins as identified and quantified by **(b)** label free proteomic analysis; one representative biological sample run in technical triplicates is shown (three additional biological samples, also run in technical triplicates and a fifth sample derived from pooled lymph from 12 individual rats are shown in Fig. 1S and **(c)** TMT proteomic analysis on the pooled lymph from twelve individual rats. List of proteins on the heat maps and their quantification is reported in Supplement Table S1. The protein ratios from TMT quantitative analysis were used to generate the heat maps after they were rescaled using a log2 transformation, such that positive values reflect fold increases (red color) and negative values reflect fold decreases (green color). PEAKS Q significance score > 10.0 was used to assess the statistically significance of the TMT heat map. In the case of LFQ analysis, only proteins which passed a selected significance statistical threshold (ANOVA, p < 0.05 and FDR < 1% for protein and peptide expression) are represented in the heat maps. **(d)** Representative comparative base chromatograms (MS1/MS2) for one pair of pre and post nodal mesenteric lymph proteomes, out of the three nanoLC-MS/MS experiments run on a Q Exactive HF quadrupole orbitrap mass spectrometer with HCD ionization mode. **(e**,**f)** Volcano plot indicating statistical significance in the fold changes observed between the pre and post nodal proteome for **(e)** LFQ and **(f)** TMT proteomics. **(g**,**h)** Views of the intensity correlation are presented for different pre- and post- nodal sample pairs. **(g)** The Pearson’s correlation score (between 0.95–0.99) indicates the high proteomic reproducibility of all pre-nodal and post-nodal samples. **(h)** The Pearson’s correlation score (between 0.71–0.72) indicates the differences between pre- and post-nodal samples. Additional parameters of mass spectrometric analysis are reported in Supplement Figs S3 and S4.

Knowledge of the lymphatic fluid protein composition in pre- and post-nodal lymph is fundamental to understanding the nodal clearance process as well as fluid homeostasis throughout the body. To measure nodal efficiency in clearing the incoming proteome, we set up cannulation of pre- and post-nodal collectors in sixteen different rats. We collected lymph from one afferent lymphatic and from the main efferent lymphatic trunk in each rat. Rats were cannulated in such a way to minimize surgical trauma and avoid consequent proteomic changes in lymph composition. We first measured total protein concentrations in the pre- and post-nodal samples, and found a statistically significant increase in protein concentration in the post-nodal fluid (Fig. 1a). These results are in agreement with previous measurements of protein concentration in human, dog, and ovine afferent and efferent lymph^12–14^. The post-nodal increase in protein concentration underscores that fluid exchange via Starling and non –Starling-related-mechanisms can occur between the lymphatic and blood compartments within lymph nodes. Under normal conditions fluid reabsorption occurs through the blood capillaries and the high endothelial venule in the draining node, serving to concentrate the post-nodal lymph^13–17^.

As a next step towards quantification of nodal filtration of the incoming proteome we performed label-free quantitative (LFQ) proteomics of pre- and post-nodal lymph samples. The amount of lymphatic fluid that can be collected is generally very small (few microliters) and variable among animals; as such we were able to perform LFQ analysis on matched pre- and post-nodal lymph samples collected from 4 individual rats (analyzed in technical triplicates), and one sample of pooled pre- and post-nodal lymph collected from 12 additional rats (analyzed in technical quadruplicates) (Fig. 1b, Supplement Fig. S1 and Supplement Tables S1 and S2). For the pooled samples we also performed a Tandem Mass Tag (TMT) isotope labeling analysis (Fig. 1c, and Supplement Tables S1 and S2). For all the samples, equal protein amounts from pre- and post-nodal lymph were reduced, alkylated, and digested in solution with trypsin followed by reverse-phase chromatographic separation and high-resolution tandem mass spectrometry analysis. Quantification was performed using the MS1 area under the curve measurement algorithm supported by the PEAKS Q module for both LFQ and TMT analysis (Bioinformatics Solution Inc., version 8.0). The comparative base chromatograms (MS1/MS2) from LFQ analysis are presented in Fig. 1d for one pair of pre- and post-nodal mesenteric lymph samples. Differences in protein abundance between pre- and post-nodal lymph are presented as unbiased fold-change heat maps (Fig. 1b,c), generated after total protein normalization (Fig. 1a). For this analysis we selected proteins that passed a statistical significance threshold (ANOVA, p < 0.05 and FDR < 0.5% for protein and peptide expression). We also generated a volcano plot showing significance levels versus fold-change in concentration for the pre- versus post-nodal samples (Fig. 1e,f). We assessed reproducibility between biological samples in this analysis using a Pearson’s correlation analysis. Correlation values between 0.95 and 0.99 were observed between the pre-nodal samples or between the post-nodal samples indicating a high degree of biological similarity (Fig. 1g), whereas significant differences (r of 0.7) were observed, indicating biological diversity when pre- versus post-nodal lymph were compared (Fig. 1h).

**Figure 2:**
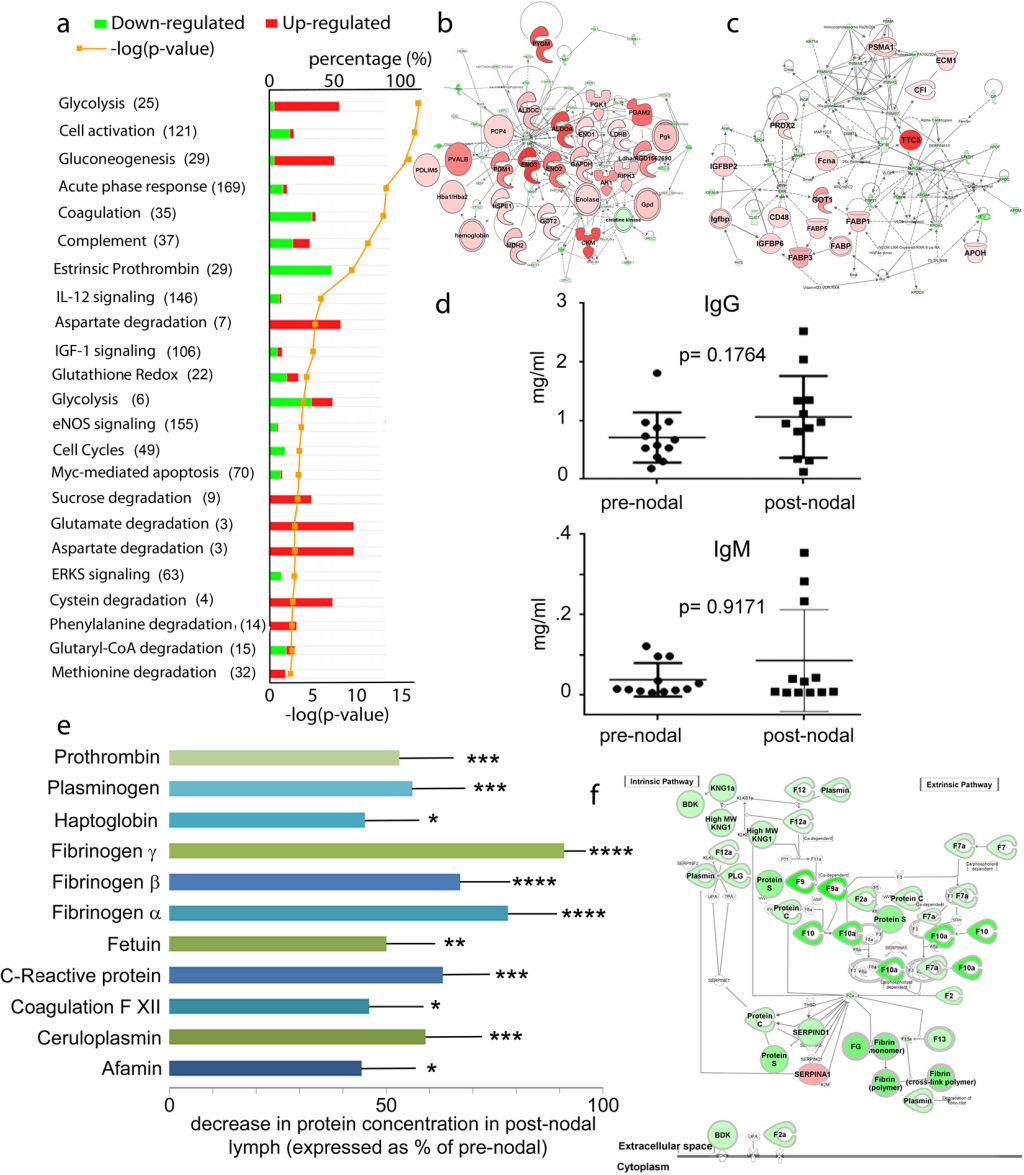
Analysis of the protein pathways up and down-regulated following nodal filtration. **(a)** Quantitative pathway analysis of proteins cleared from the pre-nodal to the post-nodal lymph following nodal transit. Proteomic analysis was performed on pre- and post-nodal lymph collected from pooled lymph from twelve rats (technical quadruplicates). Numbers in parenthesis correspond to the proteins assigned to each pathway. Bars show pathways that are up (red) or down (green) regulated in the pre- vs. post-nodal lymph. **(b**,**c)** Top-scoring networks derived from the proteins with different abundance in the pre- and post-nodal lymph generated by IPA analysis. IPA identified ten significant networks (p < 0.001) with scores above 30, which are overlaid and related to carbohydrate metabolism **(b)** and lipids, small molecules, amino acids and proteins metabolism **(c)**. The top networks are displayed in the Table S2. Green nodes depict proteins present in lower abundance in the post- vs. the pre-nodal lymph; red nodes depict proteins present in lower abundance in the pre- vs. the post-nodal lymph. Color intensity directly correlates with fold-changes, expressed as log2 (TMT pre-/post- ratio). **(d)** ELISA for total IgG and IgM pre-sent in pre- and post-nodal lymph. Each dot or square represents an individual sample, pre- or post-nodal collected from a single rat. Measurements were performed in triplicates for each sample and statistical analysis performed using a two-tailed t-test; average and standard deviation are reported. **(e)** Bar-graph reporting the percentage of nodal clearance of the most abundant lymph proteins. Data are reported as percentage of protein decrease in the post-nodal lymph, as compared to the pre-nodal, upon nodal transit. Measurements were collected from 4 individuals and 12 pooled lymph samples and statistical analysis performed using a two-tailed t-test; average and standard deviation are reported. **(f)** Proteins involved in acute phase response and coagulation pathways, as identified by IPA analysis to be statistically significant (p < 0.0001) in their fold expression calculated from the log2 (TMT ratio pre-/post-). Data were analyzed through the use of IPA (QIAGEN Inc., https://www.qiagenbioinformatics.com/products/ingenuity-pathway-analysis.

We utilized the tandem mass tag (TMT) labeling data to analyze the extent of protein clearance calculated as percentage of protein concentration the post-nodal versus pre-nodal lymph. Over 65% of the mapped proteins showed significant clearance, i.e. reduction in protein amount between pre- and post-nodal samples. Among these 10% of the proteins were cleared (reduced in concentration) to values between 2 and 20% of their original pre-nodal concentration, 40% were reduced to 50% of the pre-nodal concentration, and another 50% were reduced to less than 50% of their pre-nodal concentration (Supplement Tables S1 and S2). The remaining 35% of the mapped proteins had concentration higher in post-nodal as compared to pre-nodal lymph. Among these several proteins were associated with nodal metabolic functions (Fig. 2a–c). Other proteins enriched in post-nodal lymph are related to lymphatic transport and pumping activities (hydroxytryptamine receptor, amiloride-sensitive sodium channel protein), or are proteins known to be also found in exosomes (profilin-1, cortactin-binding protein), or are immune-related growth factors and chemokines (C-type lectin inflammatory cytokine, chemokine-like receptor 1) (Supplement Tables S1 and S2). Another major class of proteins that were slightly up-regulated in the post-nodal lymph were the immunoglobulins, due to their nodal synthesis. They up-regulation was also confirmed by ELISA (Fig. 2d, Supplement Tables S1 and S2).

To start enquiring on the efficiency of nodal protein clearance, we further analyzed proteins known to be mostly involved in maintenance of the oncotic pressure of lymphatic fluid (albumin, alpha and beta globulins, haptoglobin, and ceruloplasmin). These proteins alone contribute to around 90% of the intravascular oncotic pressure and their concentration is fundamental to avoid tissue edema. Each of these proteins is present in the pre-nodal lymph at high concentrations (>1–2 mg/ml), making them ideal candidates to follow as measures of overall nodal clearance (Fig. 2e,f, Supplement Tables S1 and S2). Altogether these proteins were efficiently cleared in the node, to level, in the post-nodal lymph, corresponding to 25 to 50% of their pre-nodal concentration. Similarly, we analyzed molecular chaperones and coagulation factors (Fig. 2e,f, Supplement Table S1 and S2). These proteins are known to be synthesized mostly in the liver, and thus, their concentrations would be mostly independent of synthesis in parenchymal organs or nodal cells. Again each of the analyzed proteins was cleared during nodal passage, with concentration reductions of close to 50% relative to their original concentrations in the pre-nodal lymph (Fig. 2f). For some selected proteins, the reduction in concentration observed by proteomic analysis was confirmed by western blotting (Supplement Fig. S2). The average coefficient of variation for the 4 replicates of the proteins used to analyze the efficiency of nodal clearance is 17.3% (standard deviation 6.1%, range 1.2% to 30.5%). The average clearance ratio for all proteins is 0.79 (standard deviation 0.05, range 0.67 to 0.92) (Supplement Table S3). The post-hoc power analysis, calculated using the observed mean clearance ratio and standard deviation, indicate that the assay is >80% empowered to detect clearance rations up to 89% using an alpha confidence error of 0.05%; all but one of the proteins fall within this range (Supplement Table S3). The remaining proteome, mostly consisting of tissue-derived antigens, was cleared to values between 70–90% of the original pre-nodal concentrations, as shown both by LFQ and TMT analysis (Supplement Table S1).

**Figure 3:**
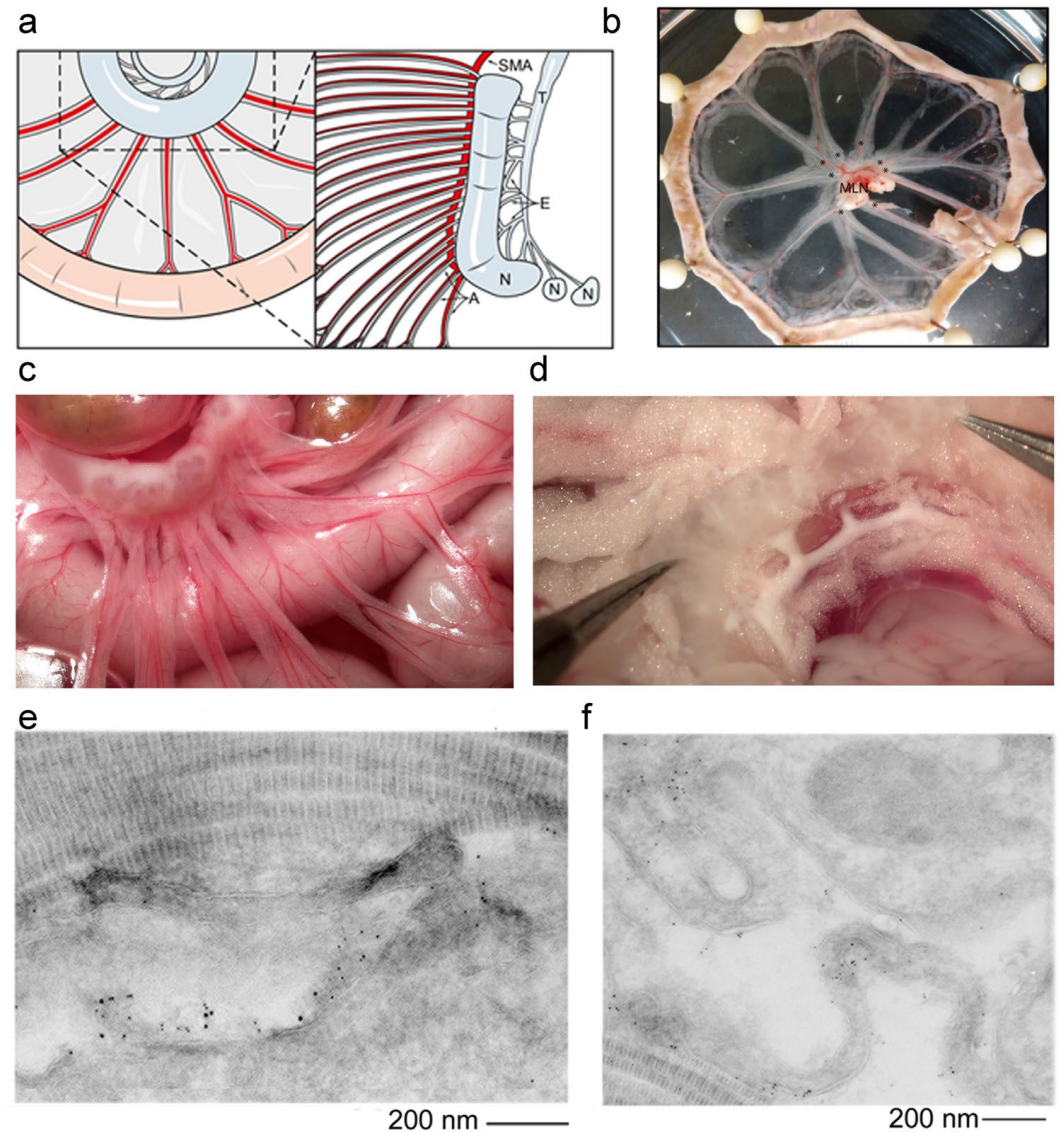
Anatomy of the nodal regions where the lymph percolates. **(a)** Schematic depicting intestinal loops with the lymphatic collectors entering and exiting the lymph node. Left Panel depicts the normal orientation of the rat ileum, mesentery and associated neurovascular bundles containing the blood (SMA) and lymphatic vessels serving the gut, the mesenteric node, and post- nodal lymphatics without the overlying adipose tissue. Right Panel depicts the arrangement of the multiple afferent pre-nodal lymphatics (A) entering the main rat mesenteric node (N), the multiple efferent post-nodal lymphatics (E) exiting the node with a complex anastomosis of the efferent lymphatics into a single main mesenteric efferent lymph trunk (T). For these experiments, we cannulated a single afferent pre-nodal lymphatic and the main mesenteric efferent lymph trunk for either collection of lymph or perfusion. **(b)** Pictures depicting intestinal loops with lymphatic collectors draining to and exiting the lymph node (center). **(c)** Picture depicting efferent lymphatic collectors entering draining lymph nodes **(d)** Picture depicting afferent collectors exiting the draining lymph nodes **(e)** Immunogold labeling with laminin (5 mm gold) and Perlecan (10 mm gold) to highlight the conduit system as the space between the central pillar of collagen and the immunogold labelled matrix proteins. **(f)** Immunogold labeling for MHC II of a dendritic cells connected to the conduit system.

The lymph proteome, entering the lymph node via the afferent lymphatics, (Fig. 3a–c) travels via two possible anatomical routes according to molecular weight of the various protein components. High molecular weight proteins (>70–80 kDa) enter the sub-cortical space and travel among the sub-cortical and then medullary sinuses before exiting through the post-nodal efferent lymphatics (Fig. 3d). The sinuses are enveloped by resident and migratory dendritic cells and macrophages. Low molecular weight proteins/peptides (<70 kDa) are thought to enter the node using an additional route: the conduit system (Fig. 3e). The conduit space spans from the subcortical space to the T-cell areas near the high endothelial venules, and is composed of laminin, perlecan and collagen fibrillary structures (Fig. 3e). Small proteins and peptides percolate through this space, and can be phagocytized by the dendritic cells, present in the T-cell areas, that directly reach into the conduit system (Fig. 3f).

**Figure 4:**
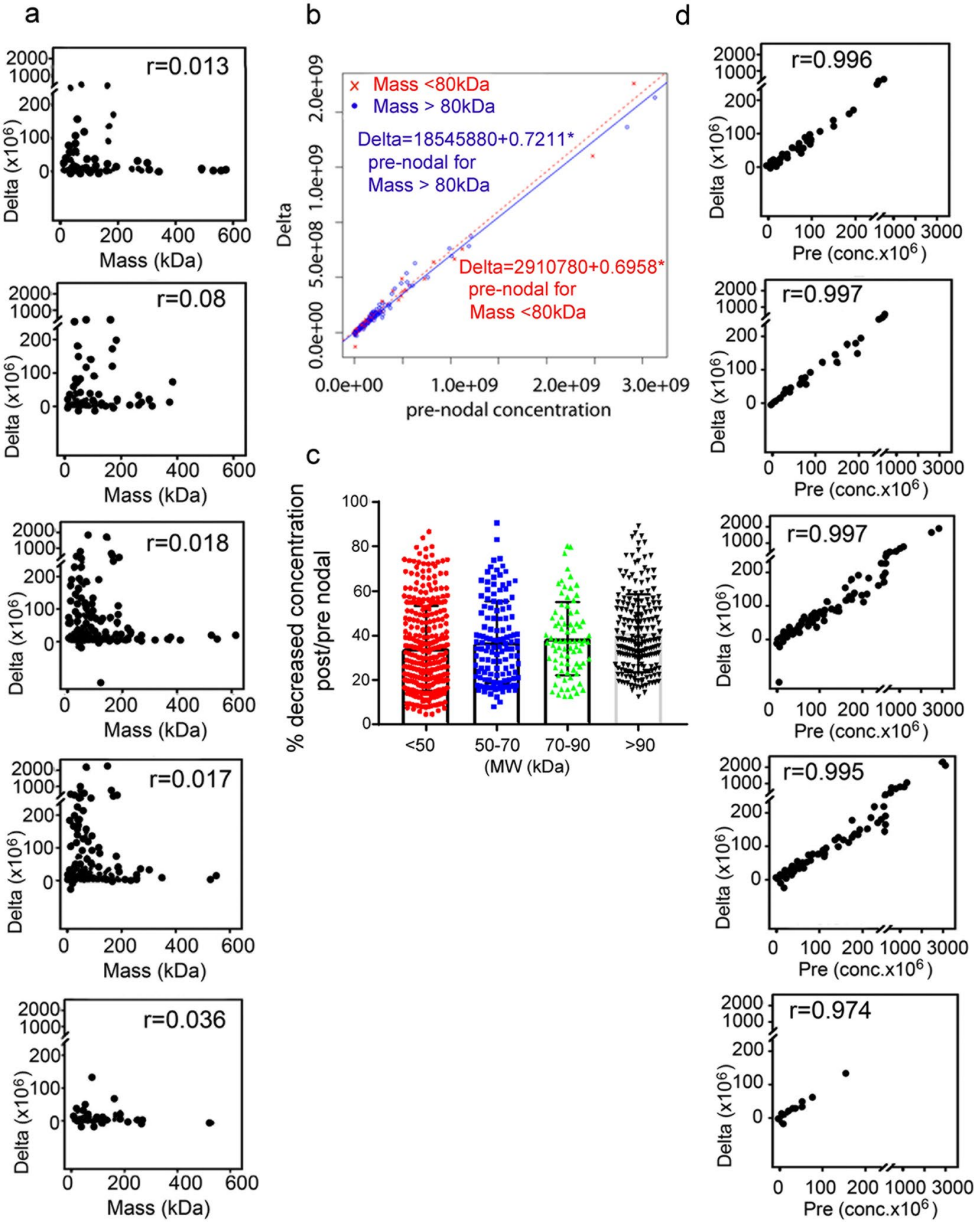
Statistical Analysis of the pre- and post-nodal proteome. **(a)** Scatter plots of delta (pre-post- changes) with protein mass for each rat (excluding albumin). **(b)** Scatter plot of delta (pre-post-) and pre-nodal concentration across all samples grouped by Mass <80 kDa and Mass >80 kDa, excluding albumin. **(c)** Analysis of pre-nodal protein clearance by the lymph node according to the protein molecular weight. The % decrease from pre- to post-nodal protein concentration, for each range of MW was calculated using the intensities of each protein as determined by quantitative TMT proteomic. **(d)** Scatter plots of delta (pre-/post- changes) with pre-nodal concentration for each rat (excluding albumin).

To determine the role played by protein molecular weight in the efficiency of protein clearance we fitted a linear model relating changes (delta) in the pre-nodal versus post-nodal protein concentrations to the protein molecular weights. We determined these changes by integrating the areas generated by LFQ analysis. This model enables us to assess the contribution of protein molecular weight to the magnitude of the changes in the pre-post concentration of the whole proteome. No significant correlation was observed between the delta concentration and protein molecular weight (Fig. 4a,b) indicating that regardless of their molecular weight proteins are effectively cleared. Because different molecular weight proteins travel through the node by different routes, efficient protein clearance does not seem to depend on nodal anatomical route. To further analyze the effect of molecular weight on protein clearance, the pre- vs post-nodal proteome was re-analyzed after division in four molecular weight ranges (Fig. 4c). When the data were plotted as % decrease in protein concentration no statistically significant differences were observed between the four groups (Fig. 4c). Overall these results confirm that regardless of the lymphatic route taken by the incoming proteome (subcortical or conduit), as dictated by the protein MW, the efficiency of clearance is similar.

As a next step we used a linear model to analyze the relationship between pre-nodal protein concentration and the magnitude of the post-nodal versus pre-nodal concentration changes. For this analysis, we used the integrated averaged area (MS1) for each pair of pre-nodal and post-nodal proteome samples. (Fig. 4d). Average areas derived from a minimum of three technical replicates as determined by LFQ analysis as shown in Supplement Table S1. We observed highly significant correlations (r = 0.99) between protein concentrations in the pre-nodal lymph and post-nodal versus pre-nodal delta concentration for lymph samples collected from four independent rats, as well for a single samples pooled from twelve pooled rats, (Fig. 4d). Thus, the higher the concentration of protein in the pre-nodal lymph, the higher would be the delta concentration effected by nodal filtration. The results from this analysis point to protein concentration in the pre-nodal lymph as a major predictor of clearance efficiency (Fig. 4d).

**Figure 5:**
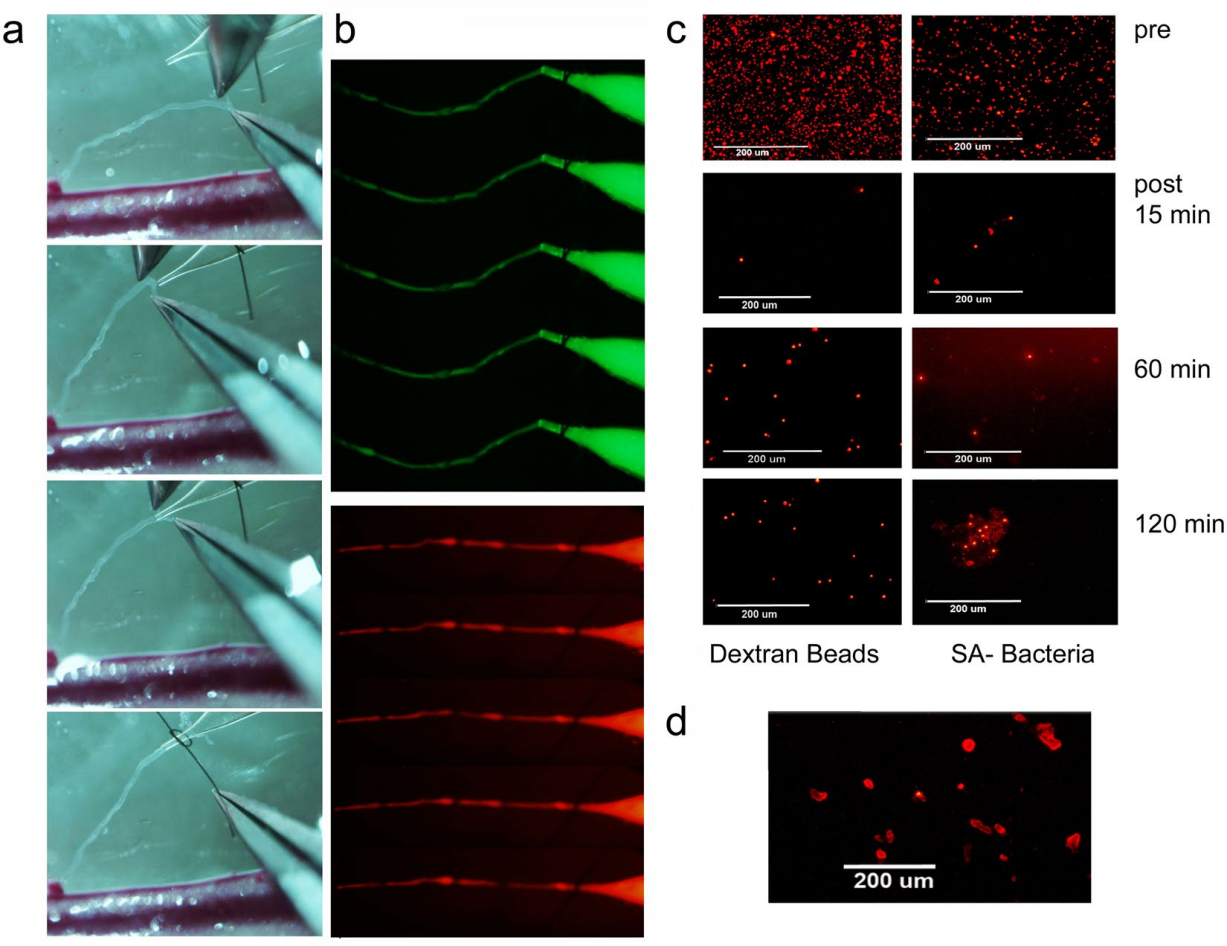
Efferent Mesenteric Lymphatic Collector Cannulation and delivery of fluorochrome-labelled proteins. **(a)** Sequential imaging depicting lymphatic preparation and cannulation **(b)** still images depicting fluorochrome-labelled proteins injected into the pre-nodal lymphatic collector. **(c)** Fluorescent dextran beads (2 μm) and *S. Aureus*, as injected in the pre-nodal lymph and retrieved in the post-nodal lymph at different time points. **(d)** Immune cells, in the post-nodal lymph following phagocytosis of fluorophore-labelled proteins and bacteria.

**Figure 6:**
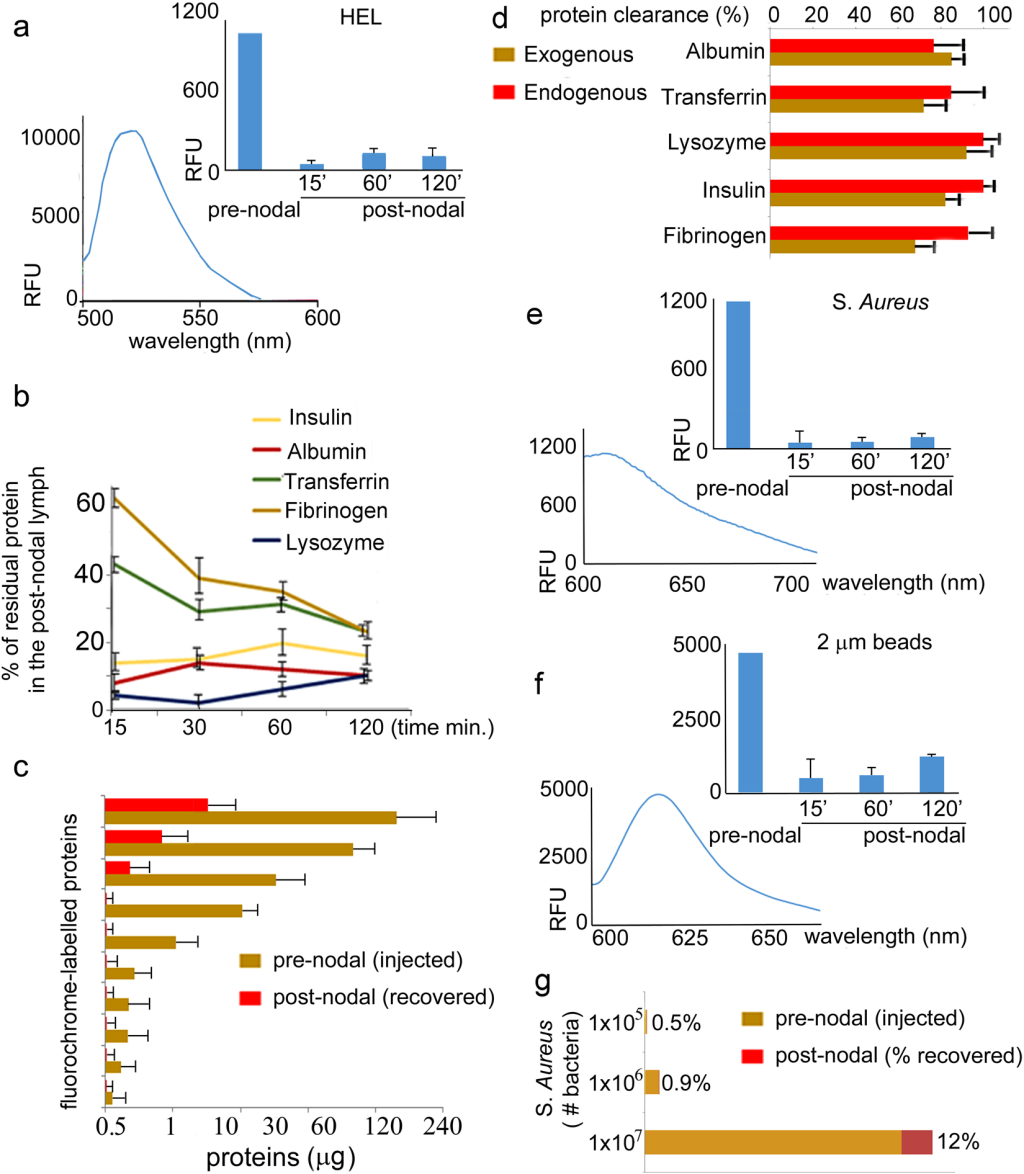
Quantitative analysis of nodal filtration. **(a)** Fluorescence trace and bar graph of HEL-FITC protein as injected in the pre-nodal lymph and as detected in the post-nodal lymph, collected at different time points. **(b)** Graph depicting percentage of fluorochrome-labelled residual proteins in the post-nodal lymph following pre-nodal injection and nodal transit for different time-points. Measurements were collected from 4 separate rats, for each time point, and statistical analysis performed using a two-tailed t-test; average and standard deviation are reported. **(c)** Bar-graphs depicting the percentage of fluorochrome-labelled proteins infused, at different concentrations, in the pre-nodal lymph and collected in the post-nodal lymph. Measurements were collected from 4 separate rats and statistical analysis performed using a two-tailed t-test; average and standard deviation are reported. **(d)** Graph depicting percentage of clearance of exogenous (fluorochrome-labelled injected proteins) and endogenous (lymph proteomic) proteins in the post-nodal lymph following nodal transit. **(e)** Fluorescence trace and bar graph of *S*. *Aureus*, as injected in the pre-nodal lymph and as detected in the post-nodal lymph, collected at different time points. Measurements were collected from 3 separate rats and statistical analysis performed using a two-tailed t-test; average and standard deviation are reported. **(f)** Fluorescence trace and bar graph of Dextran beads, as injected in the pre-nodal lymph and as detected in the post-nodal lymph, collected at different time points. Measurements were collected from 4 separate rats and statistical analysis performed using a two-tailed t-test; average and standard deviation are reported. **(g)** Bar-graph depicting percentage of *S*. *Aureus*, recovered in the post-nodal lymph following injection, of different bacterial amounts, in the pre-nodal lymph. Measurements were collected from 4 separate rats and statistical analysis performed using a two-tailed t-test; average and standard deviation are reported.

To further quantify the efficiency of nodal clearance we set up a controlled system in which known protein quantities would be infused into a cannulated 2^nd^ order afferent pre-nodal mesenteric lymphatic collector (Fig. 3a,c). The cannulated lymphatic collector was perfused with fluorophore-labelled proteins, fluorescent dextran beads, or fluorescent bacteria (Fig. 5–c), and was monitored under a microscope for periods ranging from 15 minutes to 2 hours. The tracers were infused at an intraluminal pressure of 3 cm H_2_O, similar to the average basal intra-lymphatic pressure measured in this region *in vivo*^18^ (Fig. 5a), so as to achieve an infusion rate similar to the rate we have measured previously in these lymphatics^19,20^. At the end of the infusion period a sample of post-nodal lymph was collected from the common mesenteric efferent lymph trunk. Both soluble and cellular fractions were collected in the post-nodal lymph to ensure that free as well as phagocytosed fluorescent proteins could be detected (Fig. 5d). The use of fluorophore-labelled tracers provided several important capabilities. First, we determined that injected antigens would be transported from the lymphatic collector into the lymph node without extra-vessel leakage. Second, we could confirm nodal passage, as assessed by the presence of fluorescence in the post-nodal lymph following fluorescent tracers’ injection into pre-nodal lymphatic collectors. Finally, we were able to quantify the concentration of fluorescent protein, beads, or bacteria in the pre-nodal and post-nodal samples, to directly measure the clearance efficiency (Fig. 6). Fluorescence analysis of pre-nodal versus post-nodal lymph indicated that after infusion of 0.5 to 5 μg fluorophore-labelled proteins, prepared at 125 μg/ml, only a negligible amount was seen in the post-nodal lymph. After accounting for a dilution effect (40-fold) from the non-infused afferent lymphatics, this indicated that a very large fraction (up to 80–90% of proteins) was cleared upon nodal passage (Fig. 6a,b). Upon increasing the amount of injected proteins to 30, 100 and 150 μg, the amount of residual fluorescent proteins in the post-nodal lymph increased to an 8, 25 and 30% of the injected amount respectively (Fig. 6c). Importantly the efficiencies of protein clearance observed for the exogenously injected proteins were similar to those calculated for its endogenous counterparts, as calculated from the post-nodal versus pre-nodal concentration measured by quantitative mass spectrometry (Figs 2e and 6d).

Similarly, injected fluorescent bacteria (Fig. 6e) or fluorescent Sephadex beads (Fig. 6f), were essentially completely cleared by the nodal antigen presenting cells after up to injection of 1 million particles. Above that number bacteria/beads could be seen in the post-nodal lymph (Fig. 6g).

## Discussion

Fluid flow within the interstitium always moves towards a lower pressure, and so is directed through freely open lymphatic capillaries that can easily collect fluid, molecules, cellular debris, and immune cells^2^. The lymphatic system then transports the interstitial fluid from peripheral organs to the draining lymph node by a combination of active and passive pumping mechanisms^21,22^.

As a result of lymphatic transport, lymph nodal cells are constantly exposed to self- and non-self proteomes. This exposure is a fundamental requirement for immune-surveillance^4,7,9^. There has not yet been a quantitative analysis of the efficiency of lymph nodal clearance of the incoming proteome. Several reports have described how single peripherally-injected antigens can travel to the lymph node within minutes and become processed efficiently by nodal macrophages and dendritic cells within few hours^5,6^. However, the overall clearance of a more complex pre-nodal proteome has never been addressed. This question is relevant for understanding the capacity of the lymph node system to process a composite mixture of proteins, which differ in molecular weight, concentration, and biochemical and biophysical properties, as normally occurs during both physiological and pathological conditions.

We aimed at addressing the general question, how does the complexity of the self-proteome, carried by the pre-nodal lymph change as it is cleared through the collecting lymph node? Specifically, we addressed four questions. First, how are proteins relevant to the maintenance of oncotic pressure and fluid homeostasis cleared upon nodal passage? Second, what is the efficiency of nodal removal of tissue-specific proteins? Third, is there a molecular-weight sieving effect due to the anatomical scaffold of the sub-cortical space, which excludes most proteins out of the conduit system? Finally, is the clearance process concentration dependent? To address each of these questions, we utilized a state-of-the-art, label-free quantitative (LFQ) proteomic approach as well as a TMT stable-isotope labeling approach, and applied these to analysis of pre- and post-nodal lymph samples. The combination of both technical approaches allowed several quantitative measurements.

The first question related to proteins relevant to maintenance of oncotic pressure and fluid homestasis. These proteins are synthesized in the liver and have an important function in maintaining fluid volume and intra-vessel oncotic pressure. We determined that as a group these proteins were cleared by nodal filtration between 40-to 50% of their original concentration. Albumin is the major protein involved in maintaining plasma oncotic pressure, and alpha and beta globulins are the other major contributors of the oncotic pressure. These proteins were cleared to 50% of their original pre-nodal concentration, after normalization for fluid volume. These results are in agreement with previous reports showing that the concentrations of lymph proteins are modified upon passage through the node, based on the balance of Starling and non-Starling forces acting across the lymph node^14^, including aquaporin channels^23,24^.

These previously reported experiments showed that when the afferent lymph coming into a node had a similar protein concentration as plasma, the efferent lymph leaving the node had a lower protein concentration because of the differences in hydrostatic pressures in blood versus lymph. Whereas, the opposite effect was observed (i.e. post-nodal lymph protein concentrations increased relative to pre-nodal concentrations) when the afferent lymph had a lower protein concentration than plasma, which is what is normally observed in physiological conditions^14,25,26^. Thus, the observed concentrating effect is due to the movement of water from the lymph into the bloodstream through the conduit, nodal blood capillaries, and finally the high endothelium venule. Altogether, the balance between phagocytosis of albumin, α-globulins, and β-globulins and fluid reabsorption by the HEV allows some fluid movement back into the blood circulation without compromising the lymphatic fluid osmotic pressure needed to prevent tissue edema and maintenance of molecular transport.

The second question addressed the efficiency of nodal capture of tissue-specific proteins. To assure reproducibility in the sample collection, which is required for both TMT and LFQ mass spectrometric analysis, we engineered a controlled system in which we cannulated a single mesenteric pre-nodal lymphatic collector, using an infusion pressure of 3 cm of water, similar to the pressure driving normal lymph flow. This experimental approach reports the efficacy of lymph nodal clearance, as it directly measures the ratio of proteome concentration in post-nodal versus pre-nodal lymph. Specifically, we were interested in analyzing the tissue-derived proteome before and after nodal clearance. Differently to the liver-synthesized proteins, that are important for maintenance of intra-capillary osmotic pressure and so are present at concentrations in the milligrams/ml range, the tissue proteome is present at concentrations in the range of micrograms/ml to femtograms/ml. By quantitatively comparing the pre-nodal and post-nodal concentrations of tissue-derived proteins, we determined that these proteins were significantly decreased in concentration as a result of nodal filtration. Indeed, most of the tissue-related proteins decreased by 40 to 90% of their original concentration.

To address the third and fourth questions, relating to the factors that drive differential filtration of proteins as they travel through the node, we applied a previously-developed method that uses a fluorescent albumin tracer to quantify lymph flow under physiological and pathological conditions^27^. In the previously published study, the *in vivo* clearance of Alexa 680-labelled albumin was found to be log-linear throughout a 6–7 hours measurement period with a constant lymph flow calculated from depot clearance, of −0.28 ± 0.08/min^27^. This method relies on subcutaneous injection of fluorescent albumin. This experimental setting is suitable for analysis of flow rate but not for quantitation of pre-nodal versus post-nodal concentration changes, since the amount of protein transported from the subcutaneous injection site to the lymphatic will be unknown. To overcome this problem, we performed direct lymphatic cannulation, and without altering flow pressure, we injected titrated amounts of fluorophore-labeled proteins. We used a concentration range that spanned from concentrations typical for the most abundant lymph proteins, such as albumin (2 mg/ml), to concentrations typical for tissue-specific proteins (micrograms/ml and lower). Using direct cannulation we established the efficiency of nodal clearance of the incoming proteome, and determined the efficiency of clearance for proteins present in the self and non-self-proteome. For protein concentrations up to 5 μg/ml, corresponding to the concentrations of all tissue-specific antigens, the efficiency of protein clearance upon nodal passage was up to 80–90%. For protein concentrations of 30, 100 and 150 mg/ml, corresponding to the concentrations normally observed for proteins involved in the maintenance of oncotic pressure, efficiency of protein clearance upon nodal passage was much lower, with nodal clearance efficiencies of 8, 25 and 30% respectively. Similarly, the lymph node could efficiently clear a pathogen load up to a million bacteria, but when ten million bacteria were injected in the pre-nodal lymph, around 30% of the pathogen load could be observed in the post-nodal lymph.

We then developed a linear mixed effect mathematical model, and use it to analyze the relationship between the pre-nodal and post-nodal protein concentrations. This analysis identified pre-nodal protein concentration as the major factor controlling nodal clearance, shown by the correlation between the protein concentration in the pre-nodal lymph and the change in concentration from nodal filtration. Altogether, the analysis of the whole proteome as well as analysis of infused fluorophore-labeled proteins indicated that the higher the amount of the protein in the pre-nodal lymph, the higher the amount in the post-nodal lymph.

Finally, we evaluated the importance of protein molecular weight in nodal filtration of lymph proteins. Our analysis of incoming and outgoing lymph proteomes indicated that regardless of the protein molecular weight the efficiency of clearance was similar. A large body of data in the literature, using fluorescent tracers for different molecular weight proteins, has previously determined the relationship between protein molecular weight and the nodal anatomical area where clearance occurs. Small molecules entering the lymph node can be cleared by macrophages and dendritic cells present in the sub-capsular and medullary spaces, but can also enter into the nodal conduit system^28–34^. Conduits, as present in T and B cell areas of the node, are anatomical passages formed by a central pillar or core of type 1 collagen core ensheathed by fibrinectin- and perlecan- containing basement membrane^28–31,35^. They provide a shunt between the subcortical spaces and the high endothelial venule, allowing lymph to flow from the lymphatic circulation to the blood. A molecular weight filter at the entrance of the conduit ensures that incoming pathogens cannot enter the blood stream^28–30,32^. Larger molecules and pathogens are mostly taken up by macrophages and dendritic cells present in the sub-capsular and medullary sinuses^33–35^. These studies were important in determining the link between protein molecular size and nodal distribution. Our mathematical analysis quantifying the pre-nodal vs post-nodal lymph proteins encompassed a whole range of molecular weights and indicates that proteins of all sizes are efficiently cleared by the nodal immune system, regardless of whether or not they can enter the conduit system^28–31,33,35^.

The pivotal role of lymphatic transport of antigens and immune cells is heavily underscored by the altered immune responses observed when the lymphatic circulation is impaired^11^. A few groups previously analyzed the efficiency of antigen capture by nodal antigen presenting cells, following subcutaneous injections of fluorophore-labeled proteins^5–7,32,35^. However, with dermal injection it is very difficult to quantify the amount of antigen transported to the draining node at the rate of typical lymphatic flow. Indeed, molecular transport of soluble molecules within living tissues depends on several variables including extracellular matrix porosity and composition, molecular weight of the soluble molecule, and its biophysical parameters such as molecular rigidity, globular or extended structure, and electrostatic repulsion.

In conclusion, our analysis assessed parameters affecting lymph node clearance efficiency of a complex proteome. Given that the lymph node is constantly exposed to physiological and pathological proteomes, incoming from parenchymal organs, understanding how to harness its clearance potential will be important towards design of antigen delivery systems for immunization, cancer therapy, and drug delivery, and for biomarker quantification in pathological conditions.

## Materials and Methods

### Reagents and statement on methods

Trifluoroacetic acid, acetonitrile, acetic acid, formic acid, iodacetamide and methanol were purchased from Fisher Scientific (Pittsburgh, PA). Urea, thiourea, dithiothreitol (DTT), TCEP-HCL (Tris(2-carboxyethyl) phosphine hydrochloride) iodoacetamide, ammonium bicarbonate, KCl, KH_2_PO_4_, H_3_PO_4_ and NH4CO_3_ were purchased from SIGMA (St. Louis, MO, USA) and from ThermoFisher Scientific (Waltham, MA USA). Complete Proteinase inhibitor cocktail was also purchased from Roche. Porcine trypsin (20 ug, specific activity >5,000 units/mg seq. grade modified), Lys-C (sequencing grade, 10 ug) and Glu-C, sequencing grade (10 ug) were purchased from Promega (Madison, WI). The Bradford assay was performed with the Biorad Braford assay kit (Biorad # 500006). All solutions were prepared using MilliQ water purified by an Elix 3 UV Water Purification System (Millipore, Billerica, USA) and filtered through 0.2 μm pore membrane sterile filter units (Steritop^TM^, Millipore). All methods were performed in accordance with the relevant guidelines and regulations.

### Antibodies

The following primary antibodies were used to validate by western blotting selected proteins candidates following LFQ and TMT analysis: anti fibrinogen (Catalog #NBP1-33582, Novus Biologicals); C reactive protein (Catalog # LF-MA0143 ThermoFisher); albumin (Catalog #PA1-29266, ThermoFisher); prothrombin (Catalog # NBP1-00992, Novus Biologicals); ceruloplasmin (Catalog #sc-135866 Santa Cruz).

### Fluorescence labeled proteins, bacteria and beads

Albumin from bovine serum (BSA), FITC conjugate (Catalog # A23015, ThermoFisher); Transferrin from human serum, Texas Red® Conjugate (Catalog # T2875, ThermoFisher); Fibrinogen from Human plasma, Alexa Fluor® 647 Conjugate (Catalog # F35200, ThermoFisher); Insulin-FITC conjugate labeled human recombinant (Catalog # I3661, Sigma Aldrich); Hen egg lysozyme, HEL-FITC (LS1-FC-1, Nanocs); Staphyloccocus aureus (SA particles) Alexa-Fluor 647; 0.2 and 2 um beads labeled Alexa-Fluor 647 (Molecular Probes).

### Animal procedures

Male Sprague-Dawley rats weighing 233–558 grams were used for these experiments. The rats were housed in an environmentally controlled vivarium approved by the American Association for Accreditation of Laboratory Animal Care. The animals were allowed full access to food and water with a 12-hour light dark cycle and constant temperature and humidity. All animal protocols in this study were approved by Texas A&M University Laboratory Animal Care Committee and adhered to both institutional and federal guidelines.

The rats were anesthetized by separate intra-muscular injections of Innovar-Vet (0.3 ml/kg) and diazepam (2.5 mg/kg im) and were given half-supplemental doses of Innover-Vet as needed. Innovar-Vet was a combo solution of droperidol (20 mg/ml) and fentanyl (0.4 mg/ml). The anesthetized rat was then moved inside a biosafety hood to avoid contamination in the lymph collection of perfusion procedures while a heating pad was applied as required to keep the body temperature at 37 °C. A midline laparotomy was made to open the abdominal cavity. About 8 ~ 9 cm long loop of the small intestine was exteriorized through a midline laparotomy. A section of the mesentery was gently positioned over a semicircular viewing pedestal on a vessel preparation board. A mesenteric lymphatic vessel was centered over an optical window on the preparation board. The exteriorized tissues were continuously suffused with Dulbecco’s PBS during all procedures. The animal was euthanized with a drug overdose after the procedures were finished.

### Mesenteric lymph collection

The pre- and post-nodal mesenteric lymph was collected from twelve rats in a fashion similar to what we previously described^36^. For the pre-MLN lymph collection, we first ligated the cut end of the lymphatic nearest the node to prevent post nodal lymph contamination. The cut end of the lymphatic nearest the gut wall was then cannulated and tied onto the tip of heparinized glass micropipette whose size closely matched the pre-nodal lymphatic diameter. Lymph was collected for a period of ~1–2 hour in a tube containing a complete cocktail of protease inhibitors (Roche). During the collection time the tissue was kept moist using PBS-moist gauze which could slightly dilute the interstitial fluid. The collected lymph was then centrifuged at 1,500 g for 15 minutes at 4 °C to remove the cellular components. The supernatant was then supplemented with filtered through 0.22-μm Millipore filters and stored at −80 °C before processing for proteomic and peptidomic analysis.

For collecting post-nodal lymph, a left visceral rotation was then performed to expose the post-nodal mesenteric lymphatic vessel by the bifurcation of the superior mesenteric vein from inferior vena cava on the posterior abdominal wall. We made a small opening (~0.5 × 0.5 mm) through the peritoneal membrane and the top wall of the post-nodal lymphatic with fine sharpened forceps to allow lymph to flow out. The post-nodal lymph fluid was also collected using a heparinized glass micropipette and measured with a specially modified pipet tip. The post-nodal lymph was then processed as reported above for the pre-nodal lymph.

### Pre-nodal lymphatic fluorophores tracer infusion

A 1 cm long segment of pre-nodal lymphatic vessel (80–120 μm in diameter) was exposed by carefully isolating its surrounding fat tissue with fine forceps and scissors under a dissecting microscope. For the pre-MLN lymphatic perfusion, the vessel was cut at his distal end, near the gut wall and cannulated with a heparinized glass micropipette (Heparin Sodium 1:100 in DPBS) whose size closely matched the pre-nodal lymphatic diameter. The glass micropipette was secured to the lymphatic vessel, through ligation as well as to the preparation board to provide a 3 cm H_2_O pressure head for tracer infusion. The pipet was filled with a perfusion solution of the various tracers and injected into the cannulated lymphatics for defined periods of time (15 min~2-hour perfusion).

### Lymph Proteome: LFQ analysis

#### Sample preparation

Total protein concentration for pre- and post-nodal lymph samples was determined using the Bradford micro assay. Equal protein amounts (20 μg), prepared in technical triplicates, were reduced in 15 mM TCEP.HCl (Thermo Scientific), 50 mM ammonium bicarbonate buffer, at pH 8.5, for 35 min at room temperature. The reduced proteins were further alkylated with 55 mM iodoacetamide solution, for 50 min at room temperature. Three different enzymes were used for “in solution” digestion in 50 mM ammonium bicarbonate buffer, pH 8.5, for 18 h, at 37 °C: endoproteinase Lys-C (1:50 enzyme: protein ratio); trypsin (1:20 enzyme: protein ratio) and Glu-C (1:10 enzyme: protein ratio) (sequencing grade Promega, Madison, WI, USA). The peptides mixture, extracted from all enzymatic digestions, were desalted on C18 Prep clean columns before high resolution liquid chromatography tandem mass spectrometry (LC-MS/MS).

#### nanoLC MS/MS

Technical triplicates were analyzed on a Q Exactive HF quadrupole orbitrap mass spectrometer (Thermo Fisher Scientific, Waltham, MA, USA) coupled to an Easy nLC 1000 UHPLC (Thermo Fisher Scientific) through a nanoelectrospray ion source. After equilibration with 5% acetonitrile, 0.1% formic acid, peptides were separated by a 120 min linear gradient from 4% to 30% acetonitrile in 0.1% formic acid at 400 nL/min (Optima™ LC/MS, Fisher Scientific, Pittsburgh, PA). The mass spectrometer was operated in the positive ion mode, in the data–dependent acquisition (DDA) mode. Full MS scans were obtained with a range of m/z from 300 to 1600, a mass resolution of 120,000 at m/z 200, and a target value of 1.00E+06 with the maximum injection time of 50 ms. HCD collision was performed on the 15 most significant peaks, and tandem mass spectra were acquired at a mass resolution of 30,000 at m/z 200 and a target value of 1.00E+05. Isolation of precursors was performed with a window of 1.2 Th. The dynamic exclusion time was 20 s. The normalized collision energy was 32%. We excluded precursor ions with single, unassigned, or eight and higher charge states from fragmentation selection.

#### LFQ analysis

Raw files from each technical and biological replicate were filtered, de novo sequenced and assigned with protein ID using Peaks 8.0 software (Bioinformatics Solutions, Waterloo, Canada), by searching against the rat *(Rattus norvegicus)* Swiss-Prot database (January 2017; 158,491 entries). The following search parameters were applied for LFQ analysis: trypsin, Lys-C and GluC restriction enzymes and one allowed missed cleaved at one peptide end. The parent mass tolerance was set to 5, 10 and 15 ppm (in independent searches) using monoisotopic mass, and fragment ion mass tolerance was set to 0.05 Da. For searches at 10 and 15 ppm, which retrieved peptide spectra outside the 5ppm range, each spectrum was manually inspected to confirm quality/accuracy of the MS/MS fragmentation profile. Carbamidomethyl cysteine (+57.0215 on C) was specified in PEAKS 8.0 as a fixed modification. Methionine, lysine, proline, arginine, cysteine, and asparagine oxidations (+15.99 on CKMNPR), deamidation of asparagine and glutamine (NQ-0.98) and pyro-Glu from glutamine (Q-18.01 N-term) were set as variable modifications. Data were validated using the false discovery rate (FDR) method built in PEAKS 8.0 and protein identifications were accepted if they could be characterized with a confidence score of (−10lgP) 20 and above for peptides and (−10lgP) 20 and above for proteins. However, since many peptides can still be correctly assigned with a score between 15 and 20; these additional peptides were manually analyzed for quality/accuracy of the MS/MS spectra (Supplement Figs S3 and S4). Searches were set at a minimum of 1 peptide per protein, after data were filtered for less than 0.5% FDR for peptides and less than 1% FDR for proteins identifications (p < 0.05). An independent validation of the MS/MS-based peptides and protein identification was performed with the Scaffold (version Scaffold_4.6.2, Proteome Software Inc.) using the compatible “.mzid” files of all pre- and post-nodal lymph samples exported from PEAKS 8.0. The Scaffold built in option “MuDPIT” was used to combine multiple files from biological and/or technical replicates of each pre- and post-nodal lymph samples. Peptide identifications were accepted if they could be established at greater than 95.0% probability by the Peptide Prophet algorithm with Scaffold delta-mass correction. Protein identifications were accepted if they could be established at greater than 90.0% probability and contained at least 1 identified peptide. The Protein Prophet algorithm assigned protein probabilities. Proteins that contained similar peptides and could not be differentiated based on MS/MS analysis alone were grouped to satisfy the principles of parsimony. Proteins were annotated with GO terms from NCBI (downloaded on March 2016).

#### Label-free relative peptide quantification

The label-free quantification was performed using the quantification algorithm supported by the PEAKS Q module (Bioinformatics Solution Inc., version 8.0). The data were filtered, smoothed, and aligned in retention time, followed by feature detection based on peak volume and isotopic clustering using the algorithm of PEAKS 8.0. MS/MS spectra were then extracted by the same software and used to search against the target-decoy database containing all Swiss-Prot entries for the *Rattus norvegicus* (January 2017; 158,491 entries) as described above. The *Quantitative* module (Q) of PEAKS 8.0 was used to perform the LFQ analysis using the precursor ion quantification built-in algorithm. The retention time (RT) shift was scanned between three and one minute, and since, after alignment there were no meaningful results outside a one-minute window, we performed all LFQ analysis using the 1-minute RT shift. The mass error tolerance was set at 5 ppm. The relative protein abundance was displayed as a heat map of the representative proteins of each protein group after normalization of the corresponding averaged areas (abundances) with respect to the total ion current (TIC), using the average ratio of the post- vs pre-nodal lymph concentration presented in Fig. 1a. The representative proteins were clustered if they exhibited a similar expression trend across all replicate samples. The hierarchical clustering was generated using a neighbor-joining algorithm with a Euclidean distance similarity measurement of the log2 ratios of the abundance of each sample relative to the average abundance, built-in in PEAKS 8.0 Q module (ref Peaks 8.0 textbook). In the heat map the positive values reflect fold increases (red color) and negative values reflect fold decreases (green color). Only proteins which passed a selected significance statistical threshold (ANOVA, p < 0.05 and FDR < 1% for protein and peptide expression) are shown in the representative heat maps of the LFQ output for each rat. The volcano plot was used to display the significance (score assigned by PEAKS 8.0 algorithm) versus fold-change of the quantified proteins in pre- vs post nodal lymph (ratio pre/post on x axis). The correlation plots views corresponding to the intensities recorded for the pre- and the post-nodal samples were displayed for different pre- and post- pairs together with the Pearson’s correlation score to assess the reproducibility of the LFQ experiment. A Pearson correlation score of 0.95–0.99 represents a high reproducibility for the LFQ analysis using a selected pair of pre- and post-nodal samples for each technical replicate. A similar LFQ analysis was performed on the pooled pre- and post-nodal lymph samples from 7 rats run as technical quadruplicates. Supplementary Table 1 provides the information related to all protein abundances and ratios and their corresponding PEAKS Q significance scores^37–39^.

### Lymph proteome: TMT quantitative analysis

#### Sample preparation

The 7 most abundant proteins (albumin, IgG, fibrinogen, transferrin, IgM, α_1_-anti-trypsin and haptoglobin) were depleted using the antibody-based multiple affinity removal spin cartridge (Seppro^®^ IgY-R7, Sigma). Depletion was performed using buffers provided with the kit and according to manufacturer’s instructions. The samples were then digested according to the FASP protocol using a 10 kDa molecular weight cutoff filter^40^. In brief, 90 μg of the lymph samples were mixed in the filter unit with 8 M urea, 0.1 M triethyl ammonium bicarbonate (TEAB) pH 8.0, and centrifuged at 14 000 *g* for 15 min. The proteins were reduced with 10 mM DTT for 30 min at RT, centrifuged, and alkylated with 55 mM iodoacetamide for 30 min at RT in the dark. Following centrifugation, samples were washed 3 times with urea solution, and 3 times with 50 mM TEAB, pH 8.0. Protein digestion was carried out with sequencing grade modified Trypsin (Promega) at 1/50 protease/protein (wt/wt) at 37 °C overnight. Peptides were recovered from the filter using 50 mM TEAB.

#### TMT labeling

The Tandem Mass Tag (TMT) labeling was performed according to the manufacturer’s instructions (Thermo Fisher Scientific, Rockford, IL). The TMT reagents (0.8 mg) were dissolved in 41 μl of anhydrous acetonitrile. Aliquots of the lymph tryptic digest were derivatized with TMTduplex chemical labels 126 and 127. After the labeling, reaction mixtures were incubated at room temperature for 1 hour, 8 μl of 5% hydroxylamine solution in water was added to quench the labeling reaction. The TMT-modified digest was combined into one sample and separated by high pH reversed phase chromatography on a Gemini-NH C18, 4.6 × 250 mm analytical column containing 3 μM particles; flow rate was 0.6 mL/min. The solvent consisted of 20 mM ammonium bicarbonate (pH 10) as mobile phase (A) and 20 mM ammonium bicarbonate and 75% ACN (pH 10) as mobile-phase B. Sample separation was accomplished using the following linear gradient: from 0 to 5% B in 10 min, from 5 to 50% B in 80 min, from 50 to 100% B in 10 min, and held at 100% B for an additional 10 min. 96 fractions were collected along with the LC separation and were concatenated into 24 fractions by combining fractions 1, 25, 49, 73 and so on. Samples were dried in Speed-Vac and desalted and concentrated on Thermo Scientific Pierce C18 Tip.

#### Mass spectrometry

Samples were analyzed on a Q Exactive HF quadrupole orbitrap mass spectrometer (Thermo Fisher Scientific, Waltham, MA, USA) coupled to an Easy nLC 1000 UHPLC (Thermo Fisher Scientific) through a nanoelectrospray ion source. Peptides were separated on a self-made C18 analytical column (100 µm internal diameter, x 20 cm length) packed with 2.7 µm Phenomenex Cortecs particles. After equilibration with 3 μl 5% acetonitrile 0.1% formic acid, the peptides were separated by a 120 minute linear gradient from 4% to 30% acetonitrile with 0.1% formic acid at 400nL/min. LC mobile phase solvents and sample dilutions used 0.1% formic acid in water (Buffer A) and 0.1% formic acid in acetonitrile (Buffer B) (Optima™ LC/MS, Fisher Scientific, Pittsburgh, PA). Data acquisition was performed using the instrument supplied Xcalibur™ (version 4.0) software. The mass spectrometer was operated in the positive ion mode, in the data–dependent acquisition mode. The full MS scans were obtained with a range of m/z 300 to 1600, a mass resolution of 120,000 at m/z 200, and a target value of 1.00E+06 with the maximum injection time of 50 ms. HCD collision was performed on the 15 most significant peaks, and tandem mass spectra were acquired at a mass resolution of 30,000 at m/z 200 and a target value of 1.00E+05 the maximum injection time of 100 ms. Isolation of precursors was performed with a window of 1.2 Th. The dynamic exclusion time was 20 s. The normalized collision energy was 32. We excluded precursor ions with single, unassigned, or eight and higher charge states from fragmentation selection.

#### TMT qualitative and quantitative analysis

The qualitative and quantitative analysis of MS/MS data derived from peptides labelled with TMTduplex isobaric reagents 126 (for pre-nodal lymph) and 127 (for post-nodal lymph) were performed using the built-in algorithm for TMT analysis and quantitative proteomics provided by PEAKS 8.0. Raw MS/MS files from TMT labeled peptides were filtered, de novo sequenced and assigned with protein ID using Peaks 8.0 software as described above for the LFQ analysis. Search parameters included 5 ppm peptide mass tolerance; 0.05 Da fragment tolerance and the following fixed modifications: cysteine +57.02510 (carbamidomethylation) and TMT2 for N-term peptide +225.16 and lysine +225.1629. The variable modifications were the same as those used for LFQ analysis. In addition, semitryptic peptides with up to three missed cleavages were allowed during the de novo sequencing and PEAKS ID searches. Data were validated using the FDR method built in the in PEAKS 8.0 and protein identifications were accepted if they could be characterized with a confidence score (−10lgP) 20 and above for peptides, and (−10lgP) 20 and above for proteins; a minimum of 1 peptide per protein after data were filtered for less than 0.5% FDR for peptides and less than 1% FDR for proteins identifications (p < 0.05). However, since many peptides can still be correctly assigned with a score between (−10lgP)15 and (−10lgP)20; these additional peptides were manually analyzed for quality/accuracy of the MS/MS spectra (Supplement Figs S3 and S4). The *Quantitative* module (Q) of PEAKS 8.0 was employed to calculate the isobaric tag ratios for the validated peptides and generate the protein ratios. The protein ratios are average of all peptides ratios and weighted by the number of quantified PSMs for each peptide. The protein ratios from TMT quantitative analysis were used to generate the heat maps after they were rescaled using a log2 transformation, such that positive values reflect fold increases (red color) and negative values reflect fold decreases (green color). Supplementary Table 1 provides the information related to all protein ratios and their corresponding (−10lgP) scores.

### Gene ontology, pathway enrichment and protein analysis of label-free and TMT proteomics data

Networks, functional analyses, and biochemical and cellular pathways were generated by employing the ingenuity pathway analysis (IPA; Ingenuity Systems, Redwood City, CA, USA) on the list of differentially abundant proteins extracted from both LFQ and TMT analyses (Supplementary Table 1). Specifically, for TMT analysis, the experimentally determined protein ratios, quantified using ≥ 1 peptides were used to calculate the experimental fold changes by rescaling the values using a log2 transformation, such that positive values reflect fold increases and negative values reflect fold decreases (as shown in the Supplementary Table 2 on TMT pathways analysis). For network generation, datasets containing gene identifiers (gene symbols) for >1.5 differentially abundant proteins between the pre- and post-nodal lymph were uploaded into the IPA application together with their rescaled log2 transformation of TMT protein ratios. These molecules were overlaid onto a global molecular network contained in the Ingenuity Knowledge Base. The networks of network-qualified molecules were then algorithmically generated based on their connectivity index using the built-in the IPA algorithm. A right-tailed Fisher’s exact test was used to calculate P values. The IPA analysis identified the pathways from the IPA library of canonical pathways that were most significant to the dataset (−log (p value) > 2.0). The results are presented in the Supplementary Table 2.

### Quantitative spectrophotometric analysis of fluorescent proteins

The steady state fluorescence emission spectra were collected with a RF-6000 spectrofluorophotometer (Shimadzu) using the same bandwidth (3–5 nm) for both emission and excitation wavelengths and a response time of 0.25–0.5 seconds for each scan. Fluorescence from Alexa Fluor 647 was detected using a filter with excitation wavelength of 590 nm and recorded emission scan between 600–740 nm. Fluorescence from FITC was detected using a filter with excitation wavelength of 490 nm and recorded emission scan between 500–620 nm. Fluorescence from Texas Red was detected using a filter with excitation wavelength of 596 nm and recorded emission scan between 605–740 nm. The relative fluorescence units (RFU), were used to calculate the total amount of fluorescent tracers (proteins, bacteria and beads (2 um)) in the pre-nodal and the post-nodal lymph samples.

### SDS-PAGE and Western blotting

Forty μg of total lymph proteins were run on a 5–15% gradient SDS-PAGE prior to blotting onto nitrocellulose (0.45 um) membranes. Membranes were blocked in 5% nonfat milk in TBST for 1 h, at RT, and probed with the primary antibody (1:300–1:1000 dilution) overnight at 4 °C. Odyssey secondary antibodies (1:5000) were added according to the manufacturer’s instructions (Goat anti rabbit IRDye 680 or 800, Goat anti mouse IRDye 680 or 800, Goat anti-Rat IgG IRDye 680 and the IRDye 680 universal detection reagent for anti-sheep IgG; LI-COR Biosciences, Cambridge, UK). Incubation with secondary antibodies was performed at room temperature for 2 h. Blots were imaged using an Odyssey Infrared Imaging System (LI-COR Biosciences, Cambridge, UK). The ImageJ software (https://imagej.nih.gov/ij) was used to quantify the WB and the data were normalized to the total protein/loaded well after Ponceau S staining.

### Enzyme linked immunosorbent assay (ELISA) quantitation of IgG and IgM

The total amount of IgG and IgM from the pre- and post-nodal lymph samples were quantified with the Rat IgG/IgM total ELISA Ready-SET-Go kits (Cat#88–50490–22/ 88–50540) from Ebioscience.com, following the manufacturer instructions. Briefly, each well of a 96-well ELISA plate (Nunc MaxiSorp) was coated with the capture antibody in 100 μl buffer containing 0.01 M Na_2_CO_3_ and 0.035 M NaHCO_3_, pH 9.6. The plates were kept at 2 hours at room temperature and washed 3 times with 200 μl/well PBS with 0.05% Tween 20 (PBST), followed by blocking with 200 μl/well of blocking buffer (1% BSA in PBS) for 2 hours at room temperature. Plates were then washed and incubated with 100 μl/well of pre-and post-nodal lymph (1:2000 dilution for IgM and 1:5000–1:10000 dilution for IgG) overnight at 4 °C (in quadruple replicates). The plates were then washed 3 times with PBST and incubated with 100 μl/well of developing antibody- HRP conjugate (1:3000 dilution) for 2 hours at room temperature. The plates were developed with the TMB substrate solution, and the absorbance was read at 450 nm on the ELISA plate reader (Spectramax Plus; Molecular Devices).

### Nodal Histology

Snap-frozen nodal tissue sections (4 μm) were fixed in form alcohol before hematoxylin-eosin staining.

### Ultrastructural analysis and immunogold staining

Nodal tissue was fixed with a mixture of 2% paraformaldehyde and 4% polyvinylpyrolodone in phosphate buffer 0.2 M (pH 7.4) at 4 °C. Fixed tissue was processed for ultrathin cryosectioning as described previously^41^. Immunogold labeling was performed using anti-Laminin (rabbit polyclonal; abcam clone ab11575) or rabbit polyclonal serum control at 10 μg/ml, and anti-Perlecan (rat IgG2a, clone A7L6), or rat IgG2a isotype control at 10 μg/ml. Secondary antibodies were anti rabbit coupled with 10 nm gold particles and streptavidin coupled with 5 nm gold particles. Contrast was obtained with a mixture of 2% methylcellulose (Sigma-Aldrich) and 0.4% uranylacetate (pH 4) (EMS). Samples were viewed under a CM120 Philips electron microscope (Philips).

### Statistic

Statistical analysis was performed using Windows GraphPad Prism 7.0 (GraphPad Software, La Jolla, California, USA). Numerical results are reported as mean +/−SE or +/−SDV when appropriate. Data are derived from a minimum of three independent experiments unless stated otherwise. Statistical significance of the difference between experimental groups, in instances of multiple means comparisons, was determined using one-way ANOVA, followed by the Bonferroni post hoc test. In addition, two-tailed unpaired 1-way ANOVA and two-tailed t-test were performed for western blot, ELISA and LFQ analyses. A “*P”* value less than 0.05 was considered significant.

To evaluate whether the magnitude of the pre-nodal and post-nodal change (delta) in concentrations was correlated with the pre-nodal concentration and mass the technical replicate MS1 from each rat sample (4 rats and pooled sample of 7 rats, separately) were analyzed by calculating Pearson correlations between delta and pre-concentrations, and between delta and mass. The models used to describe the correlation were described by the following relationship: (1) Delta (pre-post) ~ Beta * Pre (concentration) for each rat and pooled rat MS1 data set, stratified for each mass range, i.e. for each MW < 80 kDa and MW > 80 kDa; (2) Delta (pre-post) ~ Beta × Pre (concentration) × (Mass > 80 kDa or MW < 80 kDa).

## Electronic supplementary material

Supplementary Figures

Supplement table S2

Supplement Table S1

Supplement Table S3
