# Supplementary material for: Quantitative Profiling of the Lymph Node Clearance Capacity
Source: Sci Rep. 2018 Jul 26;8:11253. doi: 10.1038/s41598-018-29614-0 (PMC6062610; doi:10.1038/s41598-018-29614-0)
Supplement: Supplementary file 1 — Supplementary Figures [file 41598_2018_29614_MOESM1_ESM.pdf]

QUANTITATIVE PROFILING OF THE LYMPH NODE CLEARANCE CAPACITY

***Cristina C. Clement, ±Wei Wang, §Monika Dzieciatkowska, *Marco Cortese, §Kirk C. Hansen, ¶ Aniuska Becerra, *Sangeetha Thangaswamy, ± Irina Nizamutdinova, †Jee-Young Moon, ¶ Lawrence J. Stern, ±Anatoliy A. Gashev, ±David Zawieja, *Laura Santambrogio**

* Department of Pathology, † Department of Epidemiology & Population Health, Albert Einstein College of Medicine, 1300 Morris Park Avenue, New York, NY 10461
± Department of Medical Physiology, Texas A&M Health Science Center, 702 SW HK Dodgen Loop, Temple, TX 76504 **§** Department of Biochemistry and Molecular Genetics, University of Colorado Denver 12801 E 17th Ave, Aurora, CO 80045 ¶ Department of Pathology, University of Massachusetts Medical School, 368 Plantation St, Worcester, MA 01605.

Supplement Figure 1S
Heat maps of biological and technical triplicates of pre and post-nodal proteins as identified and quantified by label free proteomic analysis (LFQ). The protein ratios from LFQ quantitative analysis were used to generate the heat maps after they were rescaled using a log2 transformation, such that positive values reflect fold increases (red color) and negative values reflect fold decreases (green color). PEAKS Q significance score >10.0 was used to assess the statistically significance of the TMT heat map. Only proteins which passed a selected significance statistical threshold (ANOVA, $p<0.05$ and FDR <1% for protein and peptide expression) are represented in the heat maps. Proteomic analysis was performed on pre and post-nodal lymph collected from **a)** four separate rats (biological quadruplicates and technical replicates; rat # 1 is shown in Figure 1) as well as on **b)** pooled lymph from 7 rats (technical quadruplicates.

Supplement Figure 2S
Western blot analysis for some of the proteins reported in (d), to validate protein filtration as calculated by label free proteomics.

Supplement Figure 3S
a) Example of precursor mass error in ppm as assessed by Peaks version 8.0/8.5. **b)** Example of scatterplot of precursor m/z versus precursor mass error in ppm. **c)** Example of distribution of PEAKS peptide score with a cut off set at 25. **d)** Scatterplot of PEAKS peptide score versus precursor mass error with a cut off set at 25. **e)** Example of results for the filtration parameters and statistics for the filtered data in PEAKS **f)** Example of distribution of PEAKS peptide score with a cut off set at 20. **g)** Example of scatterplot of PEAKS peptide score versus precursor mass error with a cut off set at 20. **h)** Example of details on the RT shift and mass tolerance before and after alignment

provided by the LFQ analysis in PEAKS. **i)** Example of details on the M/Z shift distribution provided by the LFQ analysis in PEAKS.

Supplement Figure 4S

a) Quality control statistics for TMT analysis displaying the Box plot for each channel in PEAKS. b) Accumulation curve for quality control of TMT analysis in PEAKS. c) Coefficient of variance of the nodal clearance (post/pre-lymph protein concentration) across 48 proteins present in at least 3 out of 4 biological replicates and known to be the major contributors to the intravascular oncotic pressure.

Figure 1S

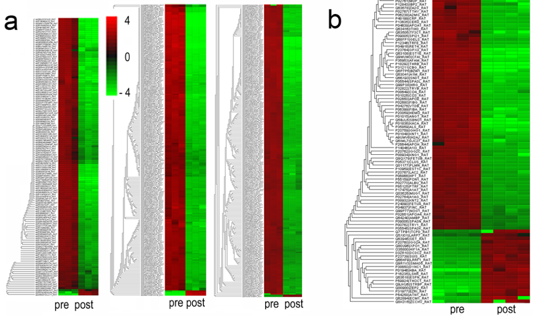

Figure 2S

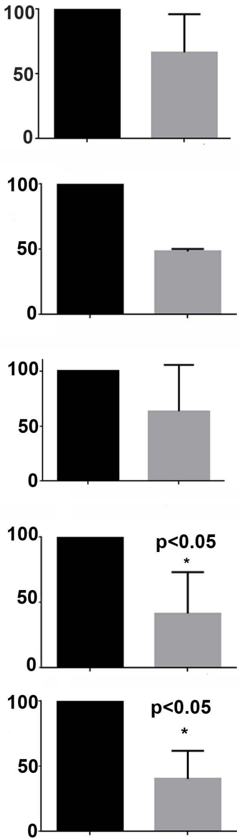

Ceruloplasmin

Prothrombin

Albumin

Fibrinogen γ

C-reactive protein

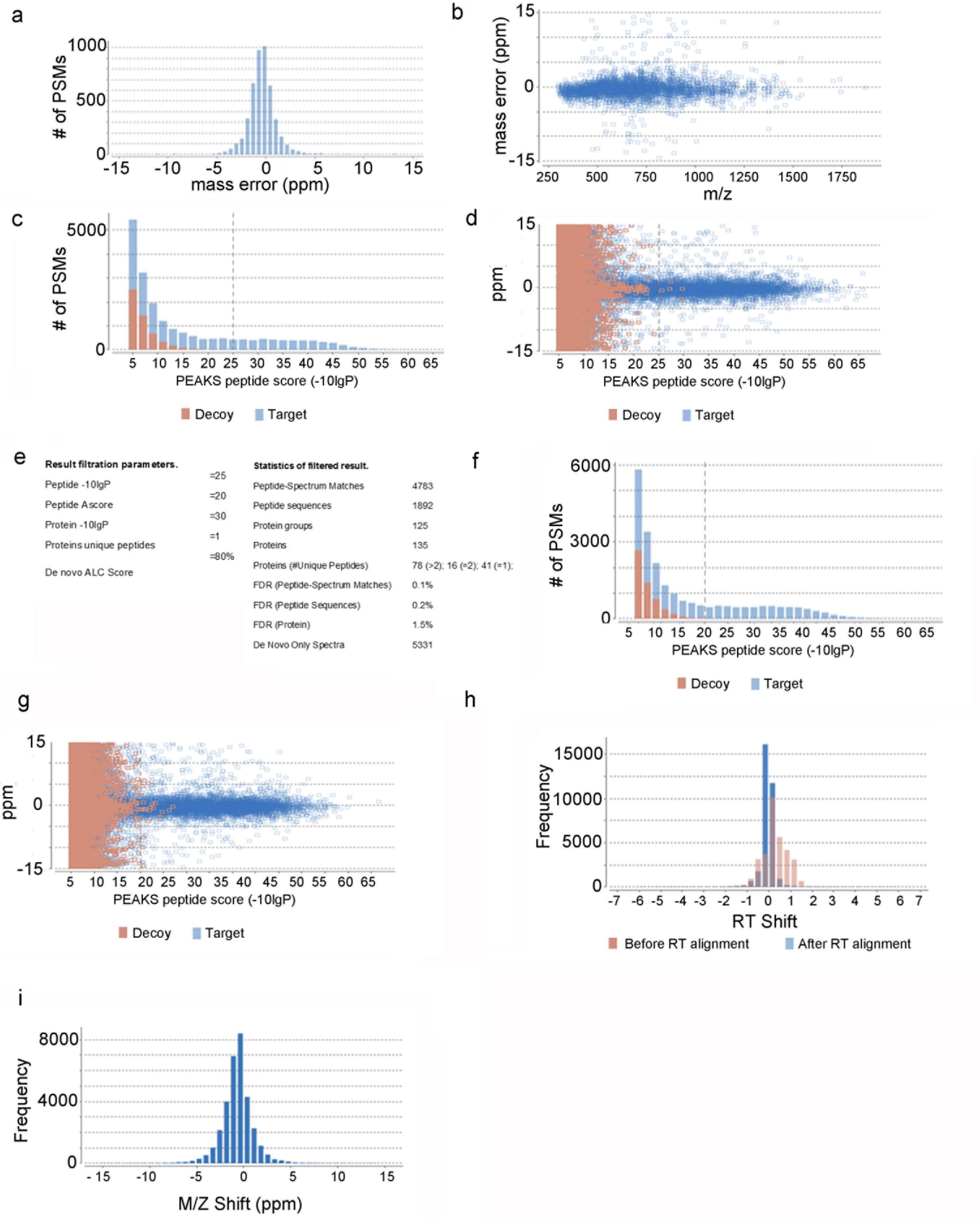
a
# of PSMs
1000
500
0
-15 -10 -5 0 5 10 15
mass error (ppm)
b
mass error (ppm)
15
0
-15
250 500 750 1000 1250 1500 1750
m/z
c
# of PSMs
5000
0
5 10 15 20 25 30 35 40 45 50 55 60 65
PEAKS peptide score (-10lgP)
Decoy Target
d
ppm
15
0
-15
5 10 15 20 25 30 35 40 45 50 55 60 65
PEAKS peptide score (-10lgP)
Decoy Target
e
Result filtration parameters.
Peptide -10lgP =25
Peptide Ascore =20
Protein -10lgP =30
Proteins unique peptides =1
De novo ALC Score =80%
Statistics of filtered result.
Peptide-Spectrum Matches 4783
Peptide sequences 1892
Protein groups 125
Proteins 135
Proteins (#Unique Peptides) 78 (>2); 16 (=2); 41 (=1);
FDR (Peptide-Spectrum Matches) 0.1%
FDR (Peptide Sequences) 0.2%
FDR (Protein) 1.5%
De Novo Only Spectra 5331
f
# of PSMs
6000
3000
0
5 10 15 20 25 30 35 40 45 50 55 60 65
PEAKS peptide score (-10lgP)
Decoy Target
g
ppm
15
0
-15
5 10 15 20 25 30 35 40 45 50 55 60 65
PEAKS peptide score (-10lgP)
Decoy Target
h
Frequency
15000
10000
5000
0
-7 -6 -5 -4 -3 -2 -1 0 1 2 3 4 5 6 7
RT Shift
Before RT alignment After RT alignment
i
Frequency
8000
4000
0
- 15 -10 -5 0 5 10 15
M/Z Shift (ppm)

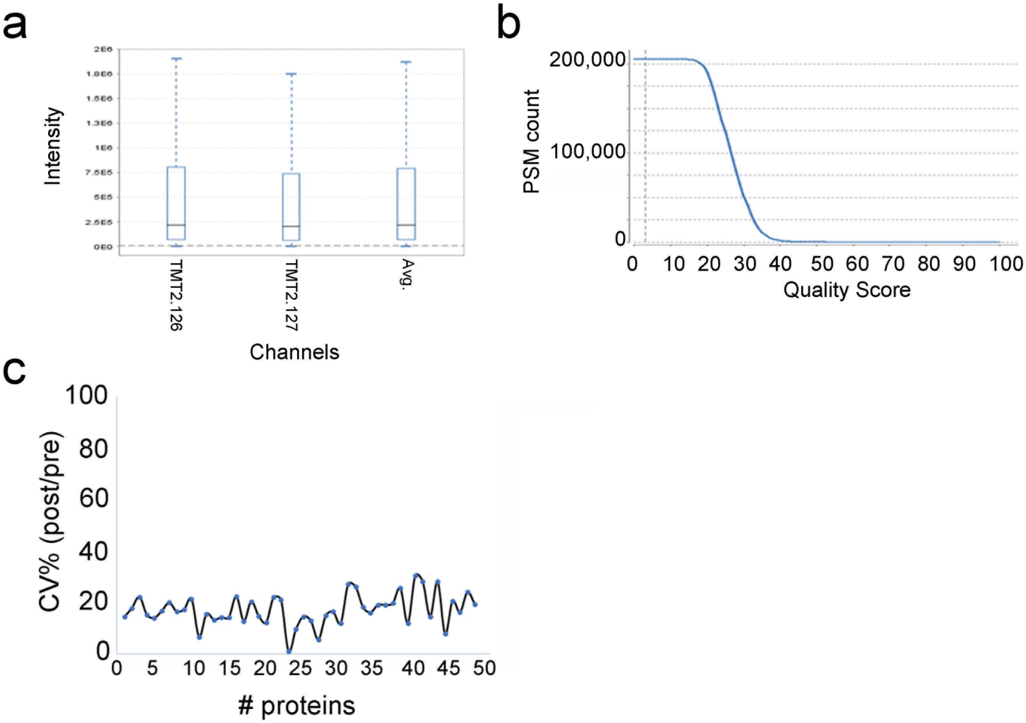

a
2E6
1.8E6
1.5E6
1.3E6
1E6
7.5E5
5E5
2.5E5
0E0
Intensity
TMT2.126
TMT2.127
Avg.
Channels
b
200,000
100,000
0
PSM count
0 10 20 30 40 50 60 70 80 90 100
Quality Score
c
100
80
60
40
20
0
CV% (post/pre)
0 5 10 15 20 25 30 35 40 45 50
proteins
